# Human Cardiospheres as a Source of Multipotent Stem and Progenitor Cells

**DOI:** 10.1155/2013/916837

**Published:** 2013-05-12

**Authors:** Lucio Barile, Mihaela Gherghiceanu, Laurenţiu M. Popescu, Tiziano Moccetti, Giuseppe Vassalli

**Affiliations:** ^1^Molecular Cardiology Laboratory and Department of Cardiology, Fondazione Cardiocentro Ticino, Via Tesserete 48, 6900 Lugano, Switzerland; ^2^Ultrastructural Pathology, “Victor Babeş” National Institute of Pathology, 99-101 Splainl Independentei, 050096 Bucharest 5, Romania; ^3^Department of Cardiology, Centre Hospitalier Universitaire Vaudois (CHUV), Avenue du Bugnon, 1011 Lausanne, Switzerland

## Abstract

Cardiospheres (CSs) are self-assembling multicellular clusters from the cellular outgrowth from cardiac explants cultured in nonadhesive substrates. They contain a core of primitive, proliferating cells, and an outer layer of mesenchymal/stromal cells and differentiating cells that express cardiomyocyte proteins and connexin 43. Because CSs contain both primitive cells and committed progenitors for the three major cell types present in the heart, that is, cardiomyocytes, endothelial cells, and smooth muscle cells, and because they are derived from percutaneous endomyocardial biopsies, they represent an attractive cell source for cardiac regeneration. In preclinical studies, CS-derived cells (CDCs) delivered to infarcted hearts resulted in improved cardiac function. CDCs have been tested safely in an initial phase-1 clinical trial in patients after myocardial infarction. Whether or not CDCs are superior to purified populations, for example, c-kit^+^ cardiac stem cells, or to gene therapy approaches for cardiac regeneration remains to be evaluated.

## 1. Introduction

Myocardial infarction (MI) and the subsequent development of congestive heart failure are the leading cause of mortality in industrialized countries. MI causes a sudden and dramatic loss of contractile heart muscle cells, or cardiomyocytes, healing with scarring. The surviving cardiomyocytes undergo hypertrophy and the heart remodels. These adaptive mechanisms are detrimental in the long run, eventually leading to pump failure. Hence, there is a pressing need for reconstituting contractile cardiac tissue after acute MI as well as in chronic heart failure, for example, in dilated cardiomyopathy. In principle, this goal could be achieved by using two general approaches, namely, by exogenous delivery of cardiomyocytes or other cell types with a potential for cardiac differentiation, or by stimulating endogenous cardiomyogenesis through appropriate small molecules or nucleic acids, either individually or in combination.

Early claims of transdifferentiation of murine bone marrow- (BM-) derived hematopoietic stem cells (HSCs) into cardiomyocytes after delivery to infarcted mouse hearts [1] were questioned by subsequent studies [2, 3]. However, these negative results did not prevent clinical studies of cell therapy for ischemic heart disease from being initiated [4–10]. A majority of the clinical trials utilized autologous BM-derived mononuclear cells delivered either into the target coronary artery or directly into the peri-infarct region [5–10]. Additional cell types that have been tested clinically in patients after MI include autologous skeletal myoblasts [11, 12], both autologous and allogeneic BM-derived mesenchymal stem cells (MSCs) [13], purified BM-derived populations such as CD133^+^ cells [4, 14], autologous BM-derived MSCs pretreated *ex vivo* with molecules that stimulate cardiomyogenic specification [15], autologous adipose tissue-derived cells [16], as well as stem and progenitor cells derived from the heart itself [17, 18]. Almost ten years after the initiation of randomized, controlled clinical trials of BM cell therapy for cardiac regeneration, it must be recognized that results have been inconsistent, and that the overall improvement of cardiac function in MI patients has been modest [19–21]. The optimal timing of cell transplantation, the delivery technique, and the most effective cell type remain to be defined. It also has been shown that reduced cell functionality in old patients and in those with advanced cardiovascular disease or comorbidities limits the benefits of autologous cells [22]. Hence, an unresolved paradox persists between robust effects of cell therapy in animal models and modest benefits in patients. In principle, cardiac-derived stem and progenitor cell populations may offer major advantages over extracardiac cell sources, as cardiac progenitors might be more prone to differentiate along the cardiomyocytic and vascular lineages and to survive in the myocardial environment [23, 24]. Most recently, two phase-1 clinical trials of autologous cardiac stem cell therapy in patients after acute MI have shown that these approaches are both safe and promising [17, 18].

## 2. *Ex Vivo* Tissue Explant Cultures, “Spheres,” and Stemness

**Figure 1:**
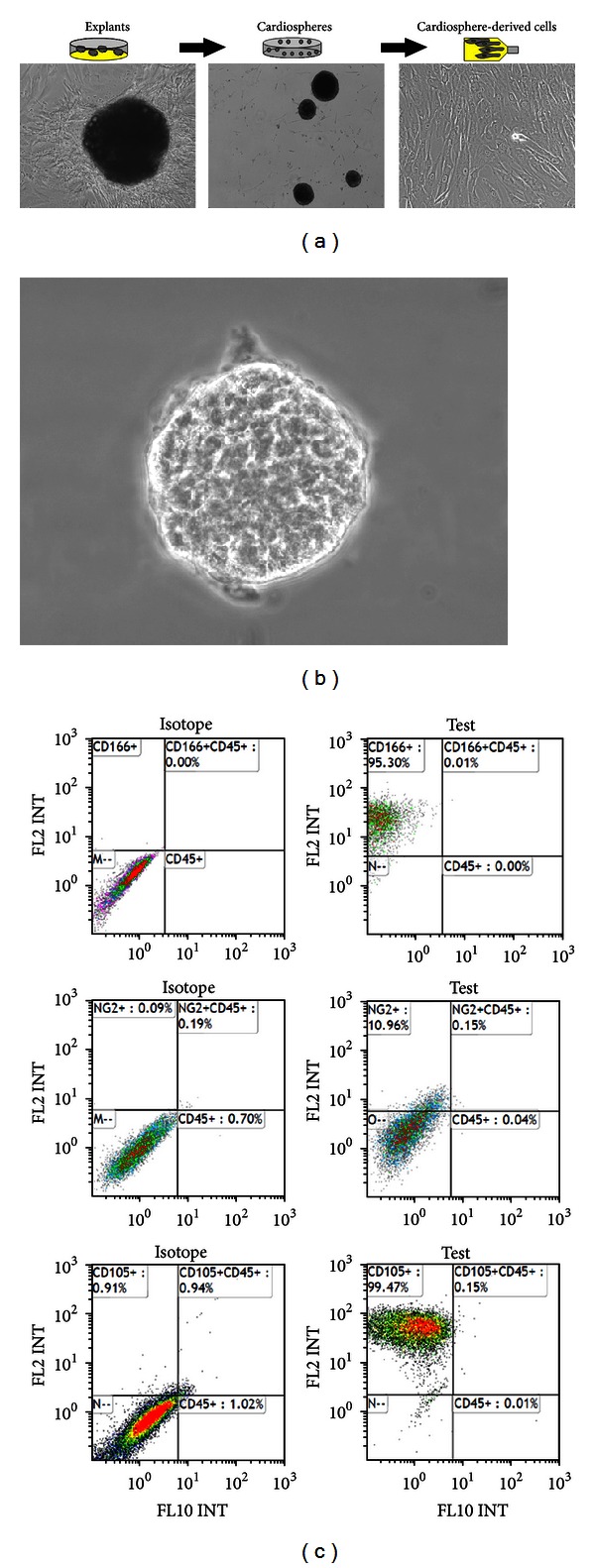
(a) Photomicrographs of a human atrial appendage specimen in the primary culture giving rise to a cellular outgrowth (left panel); CSs (middle panel); CDCs (right panel). (b) High magnification view of a human CS. (c) Flow-cytometric analysis of cell-surface marker expression by CS-forming cells (top to bottom: plots for CD45 versus CD166, CD45 versus NG2, and CD45 versus CD105 expression).

The first report that cardiac progenitor cells can be clonally expanded from murine and human myocardial biopsy specimens and form “spheres” *in vitro* came from Messina et al. [25]. Surgical atrial appendage specimens placed in the primary *ex vivo* tissue culture spontaneously shed a heterogenous cell population, the cellular outgrowth. Remarkably, we have observed that cardiac explants keep shedding cells for more than one year in the *ex vivo* culture [26], providing direct evidence for the existence of cells within the tissue explant that are able to proliferate in long term even in the absence of blood supply. When cultured in cardiosphere- (CS-) forming medium (a base medium supplemented with basic fibroblast growth factor, epidermal growth factor, cardiotrophin-1, thrombin, and B27 as a serum substitute) and the nonadhesive substrate poly-D-lysine, the cellular outgrowth gives rise to CSs (Figure 1(a)). Spheres are self-assembling, multicellular, and floating cell clusters. Sphere-forming cells may lose, in part, anchorage-dependent growth. First described in neural stem cells [27], spheres have been considered—or named, at least—as a characteristic feature of stemness. However, it is now well recognized that sphere formation is not sufficient to establish stemness [28, 29]. In fact, spheres can be either clonal or nonclonal. Decreasing cell density in culture dishes typically increases the proportion of clonal spheres, which result from clonal cell proliferation. By contrast, nonclonal spheres result from both proliferation and cell aggregation.

## 3. CSs in Rodents

The early cellular outgrowth from murine cardiac explants forms a layer of fibroblast-like cells on which numerous small round “phase-bright” cells appear with a delay of 1 to 2 weeks [26, 30]. The cellular outgrowth from neonatal mouse cardiac explants is heterogeneous and contains both hematopoietic (CD45^+^) and nonhematopoietic (CD45^−^) cells. The latter include differentiation lineage-negative (Lin^−^), c-kit^+^ (CD117) progenitor cells, endothelial cells and endothelial progenitor cells (CD31^+^ and/or CD34^+^), as well as mesenchymal/stromal progenitor cells (CD90^+^ and CD105^+^) [26, 30–34]. Davis et al. [33] recently proposed that the CD45^+^ subset within cellular outgrowths may result from a retained hematologic component, which was minimized by perfusing the heart with heparinized saline before placing the tissue explants in the culture dishes. Approximately 10% of cells shed by mouse cardiac explants during the first few days express c-kit, stem cell antigen-1 (Sca-1), the stem and progenitor cell-related antigen CD34, and the endothelial marker CD31. We have shown that CSs are composed of clonally derived cells that consist of proliferating cells primarily in their core, including a subset of c-kit^+^ cells, along with an outer sheet of early committed progenitors and differentiating cells that express cardiac, endothelial, and stromal markers. CSs from transgenic mice expressing a nuclear *lac*Z reporter gene driven by the cardiac-specific cTnI promoter exhibited lacZ expression mainly in the outer sheet [35]. By video microscopy, we have demonstrated spontaneous beating of CSs from neonatal, but not adult, mouse cardiac explants in the absence of coculture with mature cardiomyocytes [26]. Recently, Andersen et al. [34] challenged the view that CS-derived cells (CDCs) are a source of stem cells with cardiomyogenic potential. These authors showed that CSs from neonatal mice may contain small myocardial fragments that detached from the tissue explant, especially when this is not removed from the cell culture, as neonatal mouse explants become less cohesive after prolonged periods of time in culture. To address this question, we used Z/EG transgenic mice in which cardiac-specific expression of Cre-recombinase results in the excision of a *lac*Z gene and activation of expression of the second reporter gene (EGFP) in the heart [36]. Following Cre-recombinase gene transfer into the heart before the initiation of the *ex vivo* tissue culture, EGFP expression was observed in Z/EG cardiac explants but not in their cellular outgrowths, indicating that the latter lacked mature cardiomyocytes resulting from small tissue fragments detaching from the explant [26]. When cardiac explants were cultured in complete MesenCult MSC medium, a commercially available medium developed for MSC cultures, as opposed to standard media utilized in the original protocol [25], a relatively homogeneous population of plastic-adherent cells expressing hematopoietic and monocyte/macrophage markers (CD45^+^ and CD14^+^) and exhibiting MSC-like differentiation potential was obtained. At high densities, these cells formed CSs that lost adhesion to plastic and detached from culture dishes [26], even when cultured directly on plastic. These observations suggest that different experimental conditions may result in the preferential expansion of different cell populations from a heterogeneous early cellular outgrowth. Recently, Ye et al. [37] addressed the question whether the CD45^+^ cells are an essential component in CS formation. They harvested CSs from 1-week post-MI mouse hearts or from healthy hearts. CD45^+^ cells were depleted from populations of CS-forming cells by immunomagnetic beads. The depletion of CD45^+^ cells from these populations actually increased the formation of CSs compared with nondepleted populations. Purified CD45^+^ cells from CS-forming cells did not form CSs, indicating that BM-derived CD45^+^ cells are neither necessary nor sufficient for CS formation.

## 4. Human CSs Contain Both Primitive Cells and Cells Differentiating into Cardiomyocytes

We have generated human CSs from cells spontaneously shed from cultured surgical atrial appendage specimens from patients undergoing heart surgery for coronary artery disease or heart valve disease. However, CSs can also be obtained from human percutaneous endomyocardial biopsy specimens [38]. CSs placed in a new culture dish disassemble and give rise to a monolayer of CDCs that are clonogenic, can be expanded on fibronectin, and can give rise to a second generation of spheres. CS-forming cells express MSC markers [39] such as CD105 (endoglin, a part of the TGF-*β*1 receptor complex), CD13 (aminopeptidase N), and CD73 (lymphocyte-vascular adhesion-protein 2), as well as CD166 (activated leukocyte cell adhesion molecule; ALCAM; Figure 1(c)). Subsets of these cells also express NG2 chondroitin sulfate proteoglycan and CD140b (platelet-derived growth factor receptor B), which have been associated with pericytes/perivascular cells and MSCs in many tissues [40]. However, the cellular outgrowth does not express CD45 and CD34.

**Figure 2:**
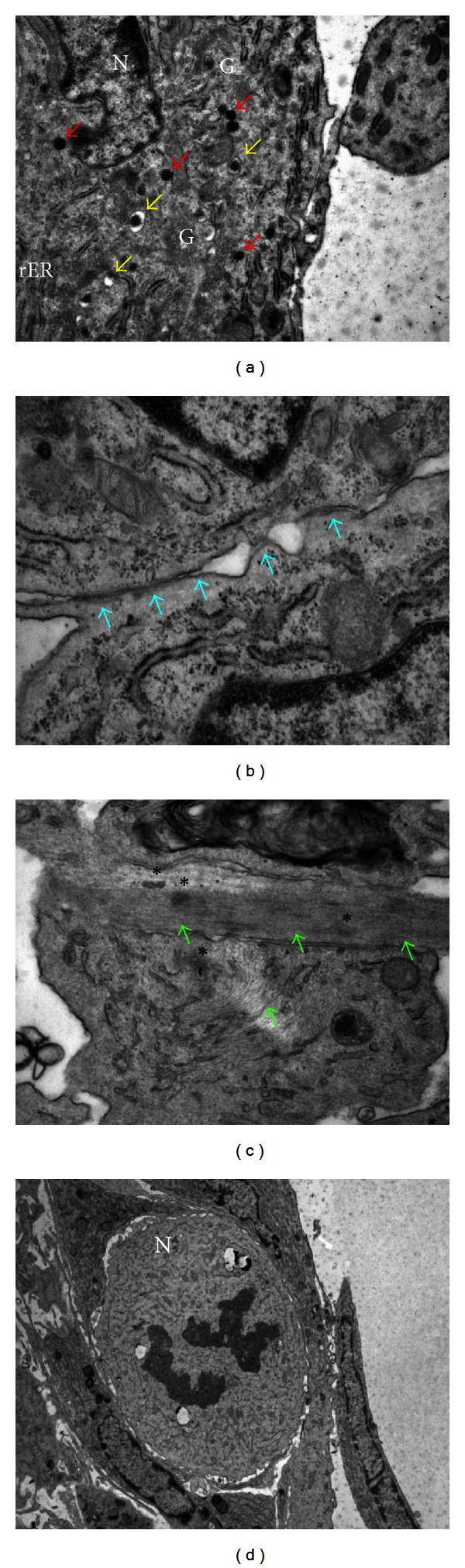
Electron-microscopical analysis of human CS ultrastructure. (a) Secretory granules (red arrows); primary lysosomes (yellow arrows). (b) Intercellular contacts (blue arrows). (c) Intracellular, unorganized thick filaments (green arrows); dense bodies (asterisks). (d) Mitosis (N, nucleus; G, Golgi apparatus; rER, rough endoplasmic reticulum).

**Figure 3:**
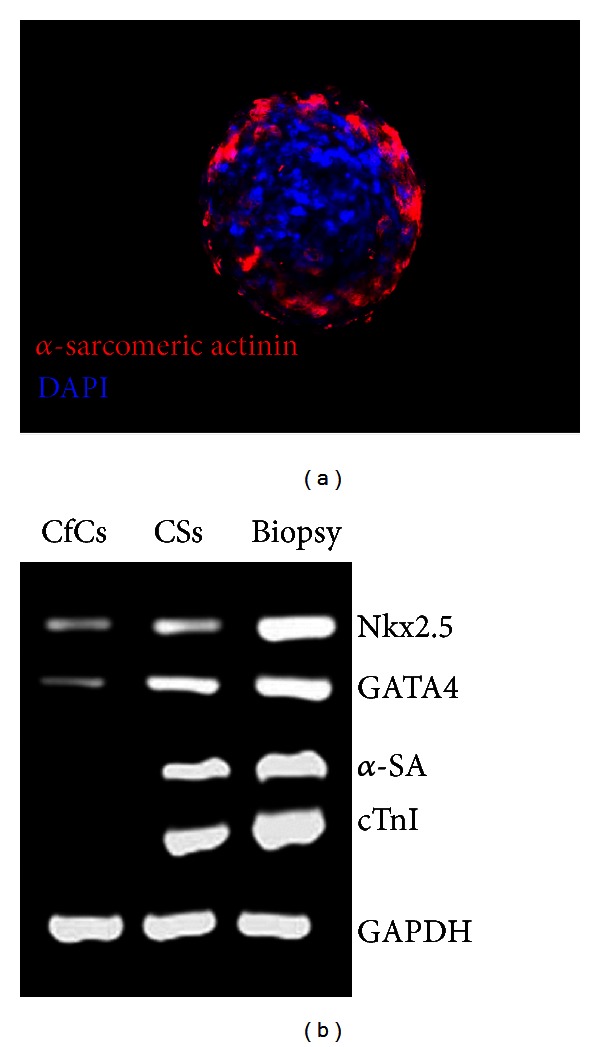
(a) Immunostaining of a human CS showing cells expressing cardiac *α*-sarcomeric actinin (red) in the outer sheet; nuclear staining with DAPI (blue). (b) PCR expression analysis of early and late cardiac genes by CS-forming cells (CfCs), CSs, and human cardiac biopsy tissue. CfCs express lower levels of early genes (Nkx2.5 and GATA4 transcription factors) compared to CSs, but no *α*-sarcomeric actinin (SA) nor cardiac troponin I (cTnI). CSs express high levels of both early and late cardiac genes.

By electron microscopy, we have provided ultrastructural evidence of the presence of secretory granules, intercellular contacts, mitotic cells, and unorganized thick filaments consistent with cardiac progenitors/precursors within human CSs (Figure 2). In line with previous studies [15, 38], we have shown that human CSs express both early (Nkx2.5 and GATA4) and late (cTnI, *α*-sarcomeric actinin) cardiac genes (Figure 3). We also have shown that cardiac troponin I and *α*-sarcomeric actinin in association with sarcomeric structures, as well as connexin 43, are detectable immunocytochemically, most abundantly in the outer layer of CSs. By contrast, cellular outgrowths from cultured cardiac tissue explants, from which CSs are derived, do not express these sarcomeric proteinsg. It has been shown that human cardiac cellular outgrowths cocultured with neonatal rat ventricular myocytes exhibit spontaneous, synchronous beating activity [35]. Moreover, differentiation of human adult CDCs could be stimulated by exposure to extremely low-frequency electromagnetic fields [41]. The CS method has also been used to enrich c-kit^+^ [42] and Sca-1^+^ cardiac cells [43].

## 5. Human Cardiospheres Recapitulate Stem Cell Niche Properties *In Vitro*

Anversa et al. [44] first postulated that CSs may recapitulate *ex vivo* several features of cardiac stem cell niches, as described *in vivo*. This notion is supported by data by Li et al. [45]. Expression of connexin 43, a gap junction protein playing a key role for the electric coupling of differentiating cardiac progenitors with the surrounding cells, suggests that the differentiated cells may serve as supporting cells for the more primitive cells. Cells self-assembled into niche-like CS structures exhibit greater proportions of c-kit^+^ cells and upregulation of embryonic genes such as SOX2 and Nanog compared to cells cultured under traditional monolayer conditions or cells dissociated from CSs. Quantitative RT-PCR and immunostaining data show increased expression of stem cell-related factors and adhesion/extracellular-matrix (ECM) molecules in CSs, including insulin-like growth factor 1 (IGF-1), histone deacetylase 2 (HDAC2), telomerase (Tert), integrin-*α*2, laminin-*β*1, and matrix metalloproteinases (MMPs) compared to the above populations not assembled in CSs. Dissociation of CSs into single cells decreases the expression of ECM and adhesion molecules, reduces the resistance of cells to oxidative stress, and abrogates the advantages of CSs in terms of *in vivo* engraftment and functional improvement after MI. Thus, CSs mimic several features of cardiac stem cell niches, including the presence of both primitive and differentiating cells and expression of ECM and adhesion molecules, which are associated with enhanced *in vivo* cell survival and cardioprotection after MI.

## 6. Human CS and CDC Therapy in Animal Models

CDCs reduced scarring after MI, increased viable myocardium, and boosted cardiac function in preclinical animal models [25, 38, 45–49]. In the initial study by Messina et al. [25], human CSs were injected into the viable myocardium bordering a freshly infarcted area in SCID mice. Eighteen days after the intervention, infarct size did not significantly differ between the CS-treated group and the PBS-injected group. However, percent fractional shortening was higher in the former group (36.85% ± 16.43% versus 17.87% ± 5.95%; *P* < 0.05). Vigorous engraftment with bands of regenerating myocardium and newly formed blood vessels were observed in the CS-treated group.

Smith et al. [38] reported that percutaneous endomyocardial biopsy specimens grown in primary culture developed CSs (in 69 of 70 patients), from which CDCs were obtained. Human CDCs were injected into the border zone of acute MIs in immunodeficient mice. CSs and CDCs expressed antigenic characteristics of stem cells at each stage of processing, as well as proteins essential for cardiac contractile and electrical function. Human CDCs cocultured with neonatal rat ventricular myocytes exhibited biophysical signatures characteristic of myocytes, including calcium transients synchronous with those of neighboring myocytes. Human CDCs injected into the border zone of MIs engrafted and migrated into the infarct zone. After 20 days, both the percentage of viable myocardium within the MI zone and left ventricular ejection fraction were greater in the CDC-treated group compared with the fibroblast-treated control group. 

Chimenti et al. [46] showed that human adult CSs and CDCs release many growth factors in culture media, which mediate both proangiogenic effects on human umbilical vein endothelial cells and antiapoptotic effects on neonatal rat ventricular myocytes *in vitro*. When transplanted into the peri-infarct zone in a SCID mouse MI model, human CDCs secreted vascular endothelial growth factor 1 (VEGF1), hepatocyte growth factor (HGF), and IGF1. These effects were associated with the upregulation of the prosurvival factor Akt, reduced the activation of caspase 3 and apoptosis, increased capillary density, and improved cardiac function. The relative contribution of the paracrine effects of the transplanted human CDCs versus their direct differentiation into cardiovascular cells was assessed by immunohistochemistry using two different antibodies raised against human-specific epitopes. The number of human-specific cells relative to overall increases in capillary density and myocardial viability indicated that direct differentiation of the transplanted cells accounted for 20% to 50% of the observed effects. These findings demonstrate that transplanted human CDCs act mainly by stimulating endogenous cardiac regeneration through paracrine mechanisms, while direct cardiac differentiation of CDCs *in situ* is also playing contributory roles.

Recently, Li et al. [47] conducted a direct comparison between different stem cell types *in vitro* for various assays of cell potency and *in vivo* for functional myocardial repair in the same mouse MI model. *In vitro*, human CDCs showed the greatest myogenic differentiation potency, the highest angiogenic potential, and relatively high production of several angiogenic and antiapoptotic factors compared with human BM-derived MSCs, adipose tissue-derived MSCs, and BM-derived mononuclear cells. *In vivo*, injection of CDCs into infarcted mouse hearts resulted in superior improvement of cardiac function, the highest cell engraftment and myogenic differentiation rates, the lowest number of apoptotic cells, and the least-abnormal heart morphology 3 weeks after treatment. The c-kit^+^ subpopulation purified from CDCs produced lower levels of paracrine factors and mediated lower functional benefits compared with unsorted CDCs. It should be noted, however, that these c-kit^+^ cells were purified from CDCs and not directly from cardiac tissue specimens, which represents a methodological difference to the recent SCIPIO trial [17]. To validate the comparison of cells from various human donors, results were verified in cells of different types derived from individual rats. These data demonstrate that CDCs have greater regeneration potential compared to other cell types currently used for cardiac repair.

## 7. Autologous versus Allogeneic CDC Therapy in Animal Models

Malliaras et al. [49] compared between syngeneic, allogeneic, and xenogeneic CDCs for cardiac regeneration. *In vitro*, CDCs expressed major histocompatibility complex (MHC) class I but not class II antigens or B7 costimulatory molecules. In mixed-lymphocyte reactions, allogeneic CDCs elicited negligible lymphocyte proliferation and inflammatory cytokine secretion. *In vivo*, syngeneic and allogeneic CDCs survived at similar levels in rat hearts 1 week after cell delivery, but few syngeneic (and even fewer allogeneic) CDCs persisted at 3 weeks. Allogeneic CDCs induced a transient, mild, and local immune reaction in the heart, without histologically evident rejection or systemic immunogenicity. Improvements in cardiac structure and function were comparable with syngeneic and allogeneic CDCs up to 6 months after cell delivery. Allogeneic CDCs stimulated endogenous regenerative mechanisms (cell cycling, recruitment of c-kit^+^ cells, and angiogenesis) and increased myocardial VEGF1, IGF1, and HGF equally with syngeneic CDCs. The persistence of benefit despite a transient survival of the transplanted cells suggested an indirect mechanism of action involving paracrine effects. These results indicated that allogeneic CDC therapy without immunosuppression was safe and improved heart function in a rat model of myocardial infarction. As such, allogeneic CDCs might obviate the limitations associated with patient-specific tissue harvesting and cell processing, suggesting that allogeneic human CDCs may represent a potential off-the-shelf product for cell heart therapy.

## 8. Clinical Testing of CDC Therapy in Patients after MI

The results of the prospective, randomised cardiosphere-derived aUtologous stem cells to reverse ventricular dySfunction (CADUCEUS) trial (registered with ClinicalTrials.gov, NCT00893360) were published recently [18]. Patients 2–4 weeks after MI (with depressed left ventricular ejection fraction of 25–45%) were enrolled at two medical centers in the USA and randomly allocated in a 2 : 1 ratio to receive CDCs (*n* = 17) or standard care (*n* = 8). For patients assigned to receive CDCs, autologous cells were grown from endomyocardial biopsy specimens. Prescribed cell doses were achieved within 36 ± 6 days (mean ± SD) and infused into the infarct-related artery 1.5–3 months after MI. The primary endpoint was proportion of patients at 6 months who died due to ventricular tachycardia, ventricular fibrillation, or sudden unexpected death or had MI after cell infusion, new cardiac tumor formation on MRI, or a major adverse cardiac event (composite of death and hospital admission for heart failure or nonfatal recurrent MI). Preliminary efficacy data were collected using cardiac magnetic resonance imaging (MRI) at 6 months. No complications were reported within 24 h of CDC infusion. By 6 months, no patients had died or developed cardiac tumors, or major adverse cardiac event in either group. Four patients (24%) in the CDC group had serious adverse events compared with one control (13%; *P* = 1.00). Compared with controls at 6 months, MRI analysis of patients treated with CDCs showed reductions in scar mass (*P* = 0.001) and increases in viable heart mass (*P* = 0.01) and regional contractility (*P* = 0.02) as well as regional systolic wall thickening (*P* = 0.015). However, changes in end-diastolic volume, end-systolic volume, and left ventricular ejection fraction did not differ between groups by 6 months. These results indicate that intracoronary infusion of autologous CDCs after MI is safe. The observed increase in viable myocardium suggests that therapeutic regeneration may have occurred.

## 9. Cell Therapy versus Secreted Factors

The demonstration of beneficial effects of cell therapy despite short-lived survival of the delivered cells [49], together with the observed trophic effects on culture media conditioned by progenitor cells [46], suggests that secreted factors may be the active component of cell therapy for cardiac regeneration. Cells communicate with each other via released molecules such as short peptides, proteins, nucleotides, and lipids that bind to surface receptors on neighboring cells. In addition, eukaryotic cells communicate with each other through the release of microparticles and exosomes in their extracellular environment. Exosomes are membrane vesicles (40–100 nm in diameter) formed by endocytosis. They are smaller than microparticles (100–1000 nm in diameter), which are released by budding of the plasma membrane (ectocytosis) [50]. Exosomes display a broad spectrum of bioactive substances on their surfaces and carry a concentrated set of proteins, lipids, and even nucleic acids that are taken up by other cells and regulate their function [51–53]. Sahoo et al. [54] reported angiogenic effects of exosomes derived from human CD34^+^ BM stem cells in isolated endothelial cells and murine models of vessel growth. In some of the *in vitro* and *in vivo* assays, the exosomes from CD34^+^ cells appeared more potent than the cells themselves. Vrijsen et al. [55] reported that exosomes mediated the angiogenic activity of media conditioned by human fetal cardiac progenitor cells *in vitro*. Timmers et al. [56] showed that injection of media conditioned by ESC-derived MSCs reduced infarct size and improved cardiac function in a pig model of ischemia/reperfusion injury, and that exosomes within the conditioned medium contained the active component. Lai et al. [57] found that exosomes secreted by MSCs similarly reduced myocardial ischemia/reperfusion (I/R) injury in mice. Barile et al. [58] recently showed that exosomes isolated from mouse cardiac progenitor cells protected H9C2 from oxidative stress by inhibiting caspase 3/7 activation *in vitro*, while also reducing cardiomyocyte apoptosis in a mouse model of myocardial I/R *in vivo*. We have provided ultrastructural evidence of exosome secretion by adult human CSs [59]. Further studies are needed to assess whether exosomes isolated from CSs are as cardioprotective as the respective cells of origin. Of note, exosomes may offer major advantages over cell transplantation for therapeutic applications. First, it might be possible to use exosomes secreted by cells from young, healthy individuals for allogeneic applications, even though this hypothesis remains to be verified. This possibility would pave the way to “off-the shelf” exosome-based therapeutic products. Second, exosomes can be stored without potentially toxic cryopreservatives at −20°C for 6 months with no loss in their biochemical activities [60]. Third, exosomes protect their contents from degradation *in vivo* [61, 62], thereby potentially preventing some of the problems associated with small soluble molecules such as cytokines, growth factors, transcription factors, and RNAs, which are rapidly degraded.

Increasing evidence suggests that exosomes may act as a vector of genetic information. Indeed, mRNAs carried by exosomes can be translated into proteins in the target cell. Accordingly, ESC-derived microvesicles were shown to reprogram hematopoietic progenitors by mRNA transfer and protein delivery [63]. MicroRNA families can be selectively secreted into the extracellular environment through exosomes [64].

## 10. Cell versus Gene Therapy

Gene therapy may provide an alternative to cell transplantation for cardiac protection and repair. Clearly, the two approaches can be used in combination by transplanting genetically engineered cells. Gene therapy has a potential for circumventing some hurdles associated with cell therapy, such as the need for *in vitro* cell expansion; however, it also has peculiar limitations, such as the need for using either viral or nonviral gene transfer vectors. Fujii et al. [65] recently showed that ultrasound-targeted gene delivery of vascular endothelial growth factor (VEGF) or stem cell factor (SCF) induced angiogenesis and improved ventricular function after MI in mice. Yaniz-Galende et al. [66] reported cardiac repair by soluble SCF gene transfer after MI via *in situ *recruitment and expansion of c-kit^+^ cells. This observation is in line with increased capillary density and reduced apoptosis in the peri-infarct area in a mouse model of tetracycline-inducible, cardiac-specific overexpression of membrane-associated SCF [67].

Exosomes carry microRNA molecules [58, 64], as mentioned above, which may play key regulatory roles in many processes such as cardiomyocyte proliferation [68], differentiation [69], hypertrophy [70], as well as aging and function [71]. Eulalio et al. [68] recently showed that exogenous administration of two microRNAs (hsa-miR-590 and hsa-miR-199a), which were identified by high-throughput functional screening for human microRNAs that promoted neonatal cardiomyocyte proliferation using a whole-genome microRNA library, markedly stimulated cardiomyocyte proliferation in both neonatal and adult rodents. After MI in mice, these microRNAs stimulated marked cardiac regeneration and almost complete recovery of cardiac functional parameters. Adenoassociated virus- (AAV-) based vectors were used to deliver microRNAs *in vivo*. Further studies are needed to evaluate whether these microRNAs likewise induce proliferation in human cardiomyocytes. Boon et al. [71] recently reported that miR-34a was induced in the aging heart and that *in vivo* silencing or genetic deletion of miR-34a reduced age-associated cardiomyocyte cell death. Moreover, miR-34a inhibition reduced cell death and fibrosis, while improving myocardial function after acute MI in mice. PNUTS, a novel direct miR-34a target, reduced telomere shortening, DNA damage responses, and cardiomyocyte apoptosis, thereby improving cardiac function after acute MI.

## 11. Conclusions

CSs have attracted great interest as an *in vitro* model of a stem cell niche-like microenvironment rich in both primitive and differentiating cells, and as a cell source for cell heart therapy. The cellular outgrowths from cultured tissue explants may enrich progenitor cells that migrate out of the explant. Moreover, both cell-cell and cell-matrix interactions within CSs may promote the specification of cardiac-resident progenitors towards cardiovascular fates. CDCs have proven safe in a phase-1 clinical trial in patients after MI, and initial results have been promising. Meanwhile, exosomes and microRNAs are emerging as alternate, cell-free strategies for cardiac protection and regeneration.
